# Imatinib-induced fulminant liver failure in chronic myeloid leukemia: role of liver transplant and second-generation tyrosine kinase inhibitors: a case report

**DOI:** 10.1186/s13256-018-1588-0

**Published:** 2018-03-10

**Authors:** Lucas Souto Nacif, Daniel R. Waisberg, Rafael Soares Pinheiro, Fabiana Roberto Lima, Vinicius Rocha-Santos, Wellington Andraus, Luiz Carneiro D’Albuquerque

**Affiliations:** 1 Liver and Gastrointestinal Transplant Division, Department of Gastroenterology, University of São Paulo School of Medicine, Rua Dr. Enéas de Carvalho Aguiar, 255-9o andar-sala 9113/9114, São Paulo, SP CEP05403-900 Brazil; 2 Department of Pathology, University of São Paulo School of Medicine, São Paulo, Brazil

**Keywords:** Acute liver failure, Liver transplantation, Organ graft, Transplantation

## Abstract

**Background:**

There is a worldwide problem of acute liver failure and mortality associated with remaining on the waiting for a liver transplant. In this study, we highlight results published in recent years by leading transplant centers in evaluating imatinib-induced acute liver failure in chronic myeloid leukemia and follow-up in liver transplantation.

**Case presentation:**

A 36-year-old brown-skinned woman (mixed Brazilian race) diagnosed 1 year earlier with chronic myeloid leukemia was started after delivery of a baby and continued for 6 months with imatinib mesylate (selective inhibitor of Bcr-Abl tyrosine kinase), which induced liver failure. We conducted a literature review using the PubMed database for articles published through September 2017, and we demonstrate a role of liver transplant in this situation for imatinib-induced liver failure. We report previously published results and a successful liver transplant after acute liver failure due to imatinib-induced in chronic myeloid leukemia treatment.

**Conclusions:**

We report a case of a successful liver transplant after acute liver failure resulting from imatinib-induced chronic myeloid leukemia treatment. The literature reveals the importance of prompt acute liver failure diagnosis and treatment with liver transplant in selected cases.

## Background

Liver transplant (LT) is an excellent therapeutic option for acute liver failure (ALF) [1]. Rapid clinical manifestation and severe hepatic injury can arise from many drugs used to treat ALF [1]. However, some situations are very unusual in clinical practice and can be related to oncological blood diseases, including chronic myeloid leukemia (CML).

Imatinib-induced ALF in a patient with CML was first described as being managed with dasatinib [2]. Even though severe hepatotoxicity may be observed in 1–4% of patients treated with imatinib, cases of fulminant liver failure are rare, with only eight known cases [2–9]. All cases but one involved patients with CML, and most of the cases were fatal [3–7]. LT was reported in four of them [2, 5, 8, 9], with one postoperative death [5]. Nilotinib was used after LT in two cases [8, 9], and Harding *et al*. first described dasatinib therapy [2]. In this report, we review the literature and report another successful case of LT for imatinib-induced liver failure.

## Case presentation

A 36-year-old brown-skinned woman (mixed Brazilian race) was admitted to our institution with jaundice, nausea, mild right upper abdominal pain, choluria, and acholia of 1 week’s duration. She had been diagnosed with CML 1 year before (during her 22nd week of pregnancy). Treatment with standard dose (400 mg/day) imatinib (Gleevec; Novartis, Basel, Switzerland) had been started after delivery and was continued for 6 months, with no adverse effects until 45 days prior to admission, when a mild elevation in liver enzymes was observed (aspartate transaminase [AST] 229 IU/L, alanine transaminase [ALT] 111 IU/L).

Imatinib was discontinued, but the patient presented on admission with ALT 1422 IU/L, AST 1690 IU/L, total bilirubin 13.08 mg/dl, prothrombin time > 50 (international normalized ratio 2.38), fibrinogen < 50 mg/dl, and factor V Leiden 11%. An abdominal ultrasound revealed no abnormalities. The results of viral screening were negative for hepatitis B (hepatitis B surface antigen, anti-hepatitis B core antibody), hepatitis C (anti-hepatitis C virus), human immunodeficiency virus (anti-HIV-I/II), cytomegalovirus (immunoglobulin G [IgG]-positive and IgM-negative), and Epstein-Barr virus. The results were also negative for autoimmune hepatitis (antimitochondrial antibody, anti-LKM1 antibody to liver kidney microsome type 1, and antinuclear antibody). The patient’s copper serum levels were normal (39 μg/dl). The laboratory findings associated with hyperammonemia and clinical manifestation of grade II/III encephalopathy led to the diagnosis of ALF.

The patient met the King’s College (O’Grady) and Clichy criteria and then underwent urgent orthotopic LT the next day. The graft weighed 0.930 kg. Total ischemic time and warm ischemic time were 6.9 hours and 35 minutes, respectively. The donor was a 40-year-old woman with brain death resulting from a ruptured brain aneurysm. The donor presented the following extended criteria: vasoactive drug use (noradrenalin 0.05 μg/kg/minute and vasopressin 0.1 μg/kg/minute), infection (nosocomial pneumonia, use of piperacillin/tazobactam for 3 days), and long intensive care unit stay (17 days). The postoperative transaminase peaks were ALT and AST of 668 IU/L and 760 IU/L, respectively. The immunosuppressive regimen featured steroids (STs) and calcineurin inhibitors as well as tacrolimus (Libbs, São Paulo, Brazil). This regimen was administered as a ST bolus during the anhepatic phase (methylprednisolone 500 mg) and was decreased to 20 mg/day and tapered to a complete stop within 6 months of LT. Tacrolimus was orally administered and maintained (0.10–0.15 mg/kg/day administered orally twice per day every 12 hours).

The patient had a good evolution during the postoperative period and was tracheally extubated on the second day. However, on the fourth postoperative day, she underwent a laparotomy day because of hemodynamic instability, coagulopathy, increased drainage through abdominal drains, and serum hemoglobin decrease after hemotransfusion without major bleeding. On the sixth postoperative day, the patient presented acute dyspnea and pulmonary congestion. Further investigation with pulmonary angiographic computed tomography ruled out pulmonary embolism. An echocardiogram showed an ejection fraction of 35% (previously 65%) without ischemic cardiac or coronary abnormalities. These symptoms were attributed to myocardiopathy secondary to systemic inflammatory response syndrome (SIRS). We excluded all other causes of cardiomyopathy (vascular, muscular, or infectious involvement, including sepsis). The patient presented a satisfactory evolution with low doses of diuretics and beta-blockers (carvedilol). She was discharged on the 25th postoperative day.

**Fig. 1:**
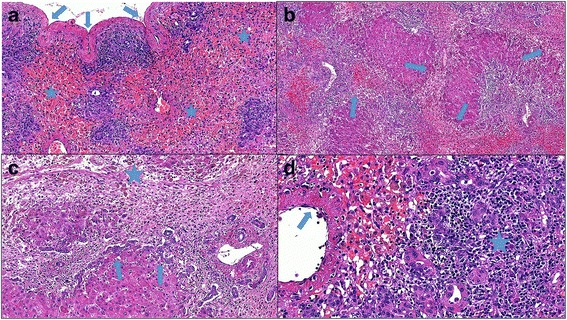
**a** A large area with multiacinar necrosis accompanied by hemorrhage and parenchymal collapse (*stars*). This resulted in liver shrinkage and wrinkling (*arrows*) of the Glisson’s capsule (hematoxylin and eosin stain, original magnification ×100). **b** Architectural distortion with bridging necrosis (*arrows*) linking central and portal zones, collapse, and hepatocellular regeneration (hematoxylin and eosin stain, original magnification ×50). **c** Periportal necrosis and central portal bridging (*star*), prominent ductular reaction (*arrows*), loose connective tissue stroma with liver cell loss, inflammation, and clusters of pigment-laden macrophages (hematoxylin and eosin stain, original magnification ×100). **d** This portal tract (on the right side of the image) is expanded by significant mononuclear inflammatory infiltrate (*star*) and ductular structures (whose cells have a variable morphology including intermediate forms). A central vein shows venulitis (*arrow*) and is located adjacent to the periportal zone because of panacinar necrosis (hematoxylin and eosin stain, original magnification ×200)

The histopathological report revealed severe acute hepatitis (Fig. 1a–d) with submassive hepatic necrosis, which is compatible with the patient’s subacute hepatic failure. Upon gross examination, the liver weighed 436 g, and the Glisson’s capsule was focally wrinkled. Histological examination showed that the more preserved parenchyma had a green to tan appearance in contrast to some irregular reddish-brown zones representing extensive necrotic areas. The left lobe was more severely damaged than the right.

Microscopically, areas of panacinar and multiacinar necrosis were intermixed with large areas of less affected parenchyma exhibiting confluent and bridging necrosis. The inflammatory infiltrate in portal tracts varied in intensity, usually with a predominance of mononuclear cells. Other findings included remarkable ductular reaction, collapsed reticulin framework, significant cholestasis, mild steatosis, hepatic enzyme induction, acidophilic bodies, and regeneration of hepatocytes. Plasma cells were not prominent. Neither granuloma formation nor eosinophil-rich infiltrate was detected. No components or any evidence of CML was found in the liver explant.

The patient remained asymptomatic for CML; nevertheless, her *BCR-ABL* transcript rose to 148% 4 months after LT. It was 0.41% prior to imatinib withdrawal. Dasatinib (Bristol-Myers Squibb, New York, NY, USA) was then introduced at 60 mg/day because of the patient’s prior history of myocardiopathy related to SIRS. No adverse effects were observed, and the patient’s *BCR-ABL* transcript reduced to 10.64% after 2 months.

At follow-up, the patient’s liver function laboratory tests (total bilirubin, transaminase levels, and coagulation profile), as well as her vascular and biliary tract (ultrasound and magnetic resonance imaging), remained normal 10 months after the LT. The patient returned to her daily routine.

### Ethical aspects

This study was approved by the institutional review board of our institution and fulfilled all of the requirements for studies in humans. It adhered to the guidelines of the 1975 Declaration of Helsinki.

## Discussion

Imatinib mesylate is a selective inhibitor of Bcr-Abl tyrosine kinase and is the first-line treatment for patients with CML without a suitable bone marrow donor or in whom transplant would be inadvisable [3]. It is also used for the systemic treatment of advanced gastrointestinal stromal tumors (GISTs) [10]. Hepatic toxicity is usually resolved with either dosage reduction or discontinuation of imatinib [3], which has been permanently required in 0.5% of patients [6]. In this scenario, second-generation tyrosine kinase inhibitors such as dasatinib, nilotinib, sunitinib, and regorafenib may be used [2, 8, 9, 11, 12].

Imatinib-related severe acute hepatitis has been described in 11 cases with favorable outcome after drug interruption (all cases) and use of prednisolone (four cases) [10]. Nine patients were female, two were male, and their age ranged from 22 to 64 years. Nine cases were CML and two were GISTs. It is interesting to note that the time between imatinib start and hepatotoxicity varied from less than 2 weeks to 77 weeks in these cases.

Table 1 presents nine cases of imatinib-related fulminant liver failure found in the literature. No ST treatment was attempted in any of these cases. The time between imatinib start and hepatotoxicity varied from 6 days to 77 weeks; nonetheless, all but two cases had at least 20 weeks of imatinib use. LT was performed in five cases.

**Table 1:** Cases of imatinib-related fulminant liver failure in the literature

Author [reference]	Year	Patient age and sex	Diagnosis	Time between imatinib start and hepatotoxicity	Treatment	Outcome
Talpaz *et al*. [3]	2002	Not reported	CML	6 days	Drug interruption	Death
Lin *et al*. [4]	2003	61 years, female	PV	7 weeks	Drug interruption	Death
Cross *et al*. [5]	2006	46 years, female	CML	77 weeks	LT	Death
Ridruejo *et al*. [6]	2007	51 years, female	CML	20 weeks	Drug interruption	Death
Thia *et al*. [7]	2008	45 years, female	CML + chronic hepatitis B	20 weeks	Drug interruption	Death
Perini *et al*. [8]	2009	47 years, female	CML	72 weeks	LT	Survived
Martínez Pascual *et al*. [9]	2010	34 years, female	CML	32 weeks	LT	Survived
Harding *et al*. [2]	2016	30 years, female	CML	20 weeks	LT	Survived
Present case	2017	36 years, female	CML	30 weeks	LT	Survived

*Abbreviations: CML* chronic myeloid leukemia, *PV* Polycythemia vera, *LT* Liver transplant

The pathogenesis of hepatotoxicity due to imatinib is unclear and may be related to hazards of drug association. Cytochrome P450 enzymes such as CYP3A4, CYP2C9, and CYP2D6 are responsible for imatinib metabolism [5, 10]. Inhibitors of CYP3A4, such as erythromycin, clarithromycin, itraconazole, roxithromycin, levonorgestrel, ethinylestradiol, cyproterone (present in some oral contraceptives), and grapefruit juice, increase imatinib concentration and can result in toxicity. In two cases of severe acute hepatitis, patients were receiving acetaminophen as well [10]. Duloxetine is metabolized by CYP1A2 and CYP2D6 pathways. These were present in one patient who ultimately underwent LT [8]. Therefore, caution should be taken when administering other hepatotoxic drugs.

Even though nilotinib and dasatinib are structurally similar to imatinib and are metabolized by the CYP3A4 pathway, previous reports have confirmed a lack of cross-intolerance. Its use appears to be safe even in cases of severe toxicity associated with imatinib. However, sunitinib and regorafenib have been associated with fatal fulminant liver failure [11] and severe acute hepatitis [12], respectively. In both cases, patients had advanced GISTs that were unresponsive to imatinib. Interestingly, no previous hepatotoxicity had been observed with this association.

Although rare, ALF in patients with oncological diseases results in ethical dilemmas. There is a scarcity of liver grafts and high mortality of patients on a waitlist. Transplant patients who may have limited survival owing to tumor recurrence might not be the best candidates. They can also suffer more than most from immunosuppression. On the other hand, fulminant liver failure is an emergency that leads to death within a few days unless the patient undergoes LT. Therefore, is it justified to abruptly shorten oncological patients’ survival if they have a favorable prognosis? There is no definitive answer. Thus, the decision must be individualized to each patient and deeply discussed by the LT recipient and the oncology teams.

## Conclusions

Imatinib-related CML cases are candidates for LT, given the favorable outcome of the few cases reported in the literature and the possibility of using equally effective medications during the post-transplant period.

## Abbreviations

ALF: Acute liver failure
ALT: Alanine transaminase
AST: Aspartate transaminase
CML: Chronic myeloid leukemia
CYP: Cytochrome P450 enzyme
GIST: Gastrointestinal stromal tumor
Ig: Immunoglobulin
LT: Liver transplant
PV: Polycythemia vera
SIRS: Systemic inflammatory response syndrome
ST: Steroid
